# Effect of Different Types of Empathy on Prosocial Behavior: Gratitude as Mediator

**DOI:** 10.3389/fpsyg.2022.768827

**Published:** 2022-02-17

**Authors:** YaLing Pang, Chao Song, Chao Ma

**Affiliations:** ^1^School of Economics and Management, Shihezi University, Shihezi, China; ^2^Faculty of Psychology and Educational Sciences, Ghent University, Ghent, Belgium; ^3^Normal School, Shihezi University, Shihezi, China

**Keywords:** prosocial behavior, gratitude, college students, mediator, empathy

## Abstract

With the development of positive psychology, prosocial behavior has received widespread attention from researchers. Some studies have shown that emotion has a significant influence on individual prosocial behavior, but little research has studied the effect of different types of empathy on college students’ prosocial behaviors. The current study examined the mediating effects of gratitude among the associations between different types of empathy (perspective-taking, fantasy, empathic concern, and personal distress) and prosocial behavior among Chinese college students. For the study, we used the Prosocial Tendency Measurement questionnaire, the Hebrew version of Interpersonal Reactivity Index-C, and The Gratitude Questionnaire that investigated 1,037 participants. The results indicated that gratitude played a mediating role between perspective-taking and prosocial behavior, fantasy and prosocial behavior, empathic concern and prosocial behavior, and personal distress and prosocial behavior, respectively. The current study contributes to a better understanding of the relationship between empathy and prosocial behavior.

## Introduction

Prosocial behavior refers to voluntary, intentional actions that benefit others (such as helping, cooperating, sharing and comforting in social interactions; Eisenberg et al., 2006, 2015). Prosocial behavior is an essential part of individuals’ social development (Wang et al., 2017), which plays a critical role in boosting happiness, improving interpersonal communication, and promoting the development of a harmonious society (Li et al., 2020). For college students, who progress to a new stage in which they live far away from their parents, prosocial behavior can be crucial to their interpersonal relationships, mental health, subjective well-being in school, and social adaptation (Meehan et al., 2019; Su et al., 2019; Son and Padilla-Walker, 2020).

Empathy is the ability to accurately recognize others’ feelings and understand the meaning of these feelings (Kalisch, 1973). One of the major points in contributing to empathy is emotioncy. Emotioncy (emotion + frequency) is defined as sense-induced emotions which can relativize cognition (Pishghadam et al., 2013). Emotioncy is of six types: Null, Auditory, Visual, Kinaesthetic, Inner, and Arch (Pishghadam, 2015). The six devised emotioncy levels are categorized into three types: avolvement, exvolvement, and involvement. In this model, emotioncy level starts with avolvement (null emotioncy), continues to exvolvement (audio emotioncy, visual emotioncy, kinesthetic emotioncy), and involvement (inner emotioncy and arch emotioncy), which includes the avolvement and exvolvement types. In this sense, each emotioncy level adds to its previous level (Miri and Pishghadam, 2021). Categorical models focus on the two distinct levels of empathy mechanisms, namely, high/cognitive and low/affective empathy. Cognitive (or high-level) empathy is defined as the ability to understand the target’s mental state by imagining how they feel, recognizing others’ emotions, and understanding others’ viewpoints (Gladstein, 1983; Hoffman, 2000; De Waal, 2008). This ability is related to perspective taking and the theory of mind (ToM) (Batson, 2009). Affective (or low-level) empathy is considered to be the automatic mimicking of other’s emotional response as one’s own, including empathic concern and personal distress (Eisenberg and Strayer, 1987; Omdahl, 1995; Batson, 2009). Cognitive empathy is the main component of empathy. It is a high-level cognitive function, such as perspective-taking and fantasy. Perspective-taking is the most cognitive in emphasis and assesses spontaneous attempts to adopt the perspectives of others and see things from their point of view, while fantasy is the tendency to identify with characters in movies, plays, and other fictional situations (Davis, 1980). Thinking about how the frustrated person is similar to oneself is an essential aspect of cognitive empathy, which can promote an individual’s prosocial behavior (Masten et al., 2011; Kim et al., 2020). Affective empathy includes empathic concern and personal distress. Empathic concern is related to experiencing warm, compassionate feelings toward people in distress, while personal distress is related to feeling others’ sadness and discomfort by observing their negative experiences (Davis, 1980).

Empathy induces an individual’s judgment and emotional experience of others’ behavior, making it easier to perceive others’ appeal for help, and promoting an individual’s prosocial behavior toward others (Eisenberg and Strayer, 1987; Omdahl, 1995; Hoffman, 2000; Stueber, 2006). The empathy-altruism hypothesis suggests that when one finds another person in an unfortunate predicament, they will have feelings of empathy and compassion for that person, which elicits the motive and behavior to help others (Batson, 1987). Empirical research has shown that empathy positively predicts prosocial behavior and that the higher level of empathy, the greater attention to the feelings and needs of others, and the more engagement in prosocial behavior (Van et al., 2018; Lindsey and Madera, 2021; Marcelo et al., 2021; Orm et al., 2021). While prior research demonstrates the relationship between empathy and prosocial behavior, researchers to date have mainly focused on children and adolescents, and little is known about how college students’ empathy affect their prosocial behavior (Zhao et al., 2019; Streit et al., 2020). Meanwhile, prior research shows that different components of empathy (perspective-taking, fantasy, empathic concern, and personal distress) may have different impacts on prosocial behavior. Linda and Anne’s (2020) research, which examined dictator, charitable giving, public goods, and trust games, showed that when empathy is divided into its four dimensions, only empathic concern has a consistent effect on prosocial behavior, while these consistent results do not hold for the other empathy dimensions. Georgiou et al.’s (2019) research indicated that affective empathy deficits cause a decrease in individuals’ prosocial behavior and an increase of antisocial behavior, while those who exhibit deficits in cognitive empathy tend to develop autistic traits, yet no reduction in prosocial behavior was found. Moreover, personal distress as a component of affective empathy has been confirmed as having a contrary impact on prosocial behavior (Batson et al., 1987; Schroeder et al., 1988; Eisenberg and Fabes, 1990; Carrera et al., 2013; Lamothe et al., 2014). Therefore, exploring the impact of different types of empathy on prosocial behavior and how different types of empathy affect college students’ prosocial behaviors may help explain the differences in prior findings and provide new ideas for college students’ prosocial behaviors.

Gratitude is a generalized tendency to recognize and respond with grateful emotion to the roles of other people’s benevolence in the positive experiences and outcomes that one obtains (McCullough et al., 2002). According to The Russian Doll model of empathy, when an individual is empathic, the affective (empathic concern and personal distress) and cognitive (perspective-taking and fantasy) components of empathy may be activated (De Waal, 2008), which will make the individual more able to stand in the position of others and perceive the efforts and losses of others, which will further stimulate the individual’s gratitude. Research has shown that individuals with higher empathy tended to have higher levels of gratitude (Worthen and Isakson, 2007; Agnieszka et al., 2020; Oriol et al., 2020). Meanwhile, the Broaden-and-Build theory of positive emotions suggests that gratitude is a positive emotion that can increase individuals’ mental flexibility, help construct psychological resources, and motivate prosocial behavior (Fredrickson, 2001, 2004). When individuals are in a positive emotional state, they will have a stronger prosocial motivation that inspires their prosocial behavior (Chen et al., 2020; Lasota et al., 2020; Yang et al., 2021). Nevertheless, no studies have examined gratitude as a potential mechanism that underlies the associations among different types of empathy and prosocial behaviors among Chinese college students.

## Materials and Methods

### Participants and Procedure

The data for this study was collected from a university in Xinjiang, China. The researchers first determined that college students were the study subjects and then contacted the teachers in the school to confirm the subjects and time of the test. Secondly, the researchers mainly conducted the pre-test training on their own teachers and students to unify the instruction of the subjects and standardize the test process. Finally, students were organized to complete the informed consent and questionnaire, which was administered in paper and pencil formal by psychology students. During the process, students majoring in psychology read instructions, explained the purpose of the survey to the subjects, and promised to keep their answers confidential. A total of 1,100 questionnaires were distributed and returned; 1,037 (94.3%) participants provided valid responses. The invalid data mainly included instances where participants did not answer carefully, for example, regular responses, and had more than a 30% missing rate. The sample included 495 males (47.7%) and 542 females (52.3%); 514 participants (49.6%) were from rural areas, and 523 participants (50.4%) were from urban areas; 720 participants (69.4%) were non-only children, and 317 participants (30.6%) were only children.

### Measures

#### Prosocial Tendencies Measure

The Prosocial Tendencies Measure (PTM) is a self-reported questionnaire developed by Carlo and Randall (2002), and translated and revised by Kou et al. (2007), which has a 26-item measure on six subscales that include public (e.g., “I prefer to help others in many public places”), anonymous (e.g., “I prefer to donate anonymously”), altruistic (e.g., “I devote my time and energy to volunteering, not to get more rewards”), compliant (e.g., “When people ask me for help, I seldom refuse”), emotional (e.g., “I’m more likely to try my best to help others when they’re emotional”) and urgent prosocial tendencies (e.g., “I tend to help people who are really in trouble and need help”). Participants indicate their agreement using a five-point Likert-type scale ranging from 1 (strongly disagree) to five (strongly agree). The higher the score of scale, the more pronounced the prosocial behavior. The PTM has been shown to have good reliability and validity in various studies and is an easily understood instrument for assessing the empathy of Chinese college students (Kou et al., 2007). In the current study, the internal consistency coefficient (Cronbach’s alpha) was 0.83.

#### The Hebrew Version of Interpersonal Reactivity Index-C

The Interpersonal Reactivity Index (IRI) is a self-reported questionnaire developed by Davis (1980), and translated and revised by Wu and Zhan (1987). The scale has 22 items on four subscales. There are two cognitive subscales: perspective-taking, the ability to adopt another’s psychological perspective and point of view (e.g., “I believe there are two sides to every problem, so I try to look at it from different perspectives”); and fantasy, the tendency to identify with characters in films and literature (e.g., “It’s rare for me to devote myself to a good book or a movie”). There are two emotional subscales: empathic concern, feelings of compassion, concern and care toward others (e.g., “For those less fortunate than me, I often feel soft-hearted and caring”); and personal distress, feelings of sadness and distress shown by the subject upon observing other people’s negative experiences (e.g., “In an emergency, I feel worried, scared and uneasy”). Participants indicate their agreement using a five-point Likert-type scale ranging from 1 (strongly disagree) to five (strongly agree). Subscale scores can be obtained by summing responses to items in each of the four subscales. The internal consistency coefficients (Cronbach’s alpha) of the four dimensional scales were 0.81, 0.79, 0.85, and 0.83.

#### The Gratitude Questionnaire-6

The Gratitude Questionnaire, developed by McCullough et al. (2002) and revised by Li et al. (2012), consists of six items, e.g., “There are so many things in life that I feel grateful for.” The average score of the six items was calculated using a seven-point Likert scale (1 = strongly disagree, 7 = strongly agree). Higher scores on the GQ-6 represent individuals with more intense levels of gratitude. The internal consistency (Cronbach’s alpha) in the current study was 0.82.

### Data Analysis

SPSS version 23.0 was used to explore the correlation analysis among the four components of empathy, gratitude, and prosocial behavior. Mediation model analysis was estimated in Mplus 7.4. We evaluated the model using model fit indices including the (a) comparative fit index (CFI) of more than 0.90 and, ideally, higher than 0.95; (b) root mean square error of approximation (RMSEA) of less than 0.08; and (c) standardized root mean square residual (SRMR) statistic of less than 0.08 (Steiger, 1990; Hu and Bentler, 1999). Meanwhile, conditional indirect effects were evaluated using a bias-corrected 95% confidence interval based on 5,000 bootstrap resamples with replacement. If the 95% confidence interval includes 0, then the indirect effect is not significant at the.05 level; if 0 is not in the interval, then the indirect effect is statistically significant at the 0.05 level (Hayes, 2013).

## Results

### Descriptive Statistics and Correlation Analysis

The result of the correlation analysis of all variables is shown in Table 1. Perspective-taking, fantasy, and empathic concern are positively correlated with gratitude, respectively, while personal distress is negatively correlated with gratitude. Perspective-taking, fantasy, empathic concern, and personal distress are positively correlated with prosocial behavior, respectively.

**TABLE 1:** Descriptive statistics and correlations for key variables.

	*M*	*SD*	1	2	3	4	5	6
1. Perspective-taking	3.63	0.66	1					
2. Fantasy	3.51	0.58	0.33**	1				
3. Empathic concern	3.55	0.59	0.14**	0.37**	1			
4. Personal distress	3.23	0.80	0.30**	0.20**	−0.07**	1		
5. Gratitude	0.91	0.16	0.25**	0.39**	0.45**	−0.06**	1	
6. Prosocial behavior	3.74	0.60	0.46**	0.25**	0.26**	0.21**	0.29**	1

**p < 0.01.

### Testing for the Mediation Model

**FIGURE 1:**
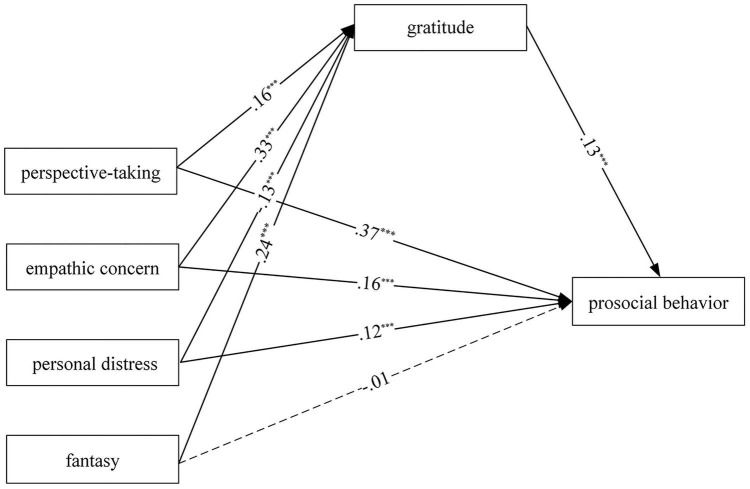
The mediation model. Path coefficients are standardized coefficients. ***p < 0.001.

Findings are presented in Figure 1. Common method bias tests show that the mediation model fits the data well [χ^2^(4) = 8.10, CFI = 0.95, RMSEA = 0.04, SRMR = 0.02]. These findings show that perspective-taking positively predicted gratitude (β = 0.16, *p* < 0.001); perspective-taking and gratitude positively predicted prosocial behavior (β = 0.37, *p* < 0.001; β = 0.13, *p* < 0.001); empathic concern positively predicted gratitude (β = 0.33, *p* < 0.001); empathic concern and gratitude positively predicted prosocial behavior (β = 0.16, *p* < 0.001; β = 0.13, *p* < 0.001); personal distress negatively predicted gratitude (β = −0.13, *p* < 0.001); personal distress and gratitude positively predicted prosocial behavior (β = 0.12, *p* < 0.001; β = 0.13, *p* < 0.001); fantasy positively predicted gratitude (β = 0.24, *p* < 0.001); gratitude positively predicted prosocial behavior (β = 0.13, *p* < 0.001), while fantasy predicted prosocial behavior’s path is not significant (*p* > 0.05).

Further, the bootstrap procedure was used to test the significance of the mediating effects. The mediating effects and the 95% confidence intervals are presented in Table 2, showing that each indirect effect was significant. The results also showed that gratitude mediates the association between perspective-taking and prosocial behavior, empathic and prosocial behavior, personal distress and prosocial behavior, fantasy and prosocial behavior, respectively.

**TABLE 2:** Bootstrap analyses of the magnitude and statistical significance of indirect effect.

	Indirect effects	SE	95% CI
1 Perspective-taking→gratitude→prosocial behavior	0.02	0.01	(0.01, 0.04)
2 Empathic concern→gratitude→prosocial behavior	0.04	0.01	(0.02, 0.07)
3 Personal distress→gratitude→prosocial behavior	−0.02	0.01	(−0.03, −0.01)
4 Fantasy→gratitude→prosocial behavior	0.03	0.01	(0.01, 0.05)

## Discussion

Prosocial behavior can reduce college students’ depressive symptoms and improve their interpersonal relationships, subjective well-being, and life satisfaction (Davis et al., 2016; Mason et al., 2019). The present study demonstrated that different components of empathy (perspective-taking, fantasy, empathic concern, and personal distress) have different impacts on prosocial behavior. The specific discussion is as follows:

We found that perspective-taking could positively predict prosocial behaviors of college students in China, which is consistent with previous research (Ding and Song, 2017; Chen et al., 2020; Lasota et al., 2020). A possible reason for this outcome is that perspective-taking enables an individual to make flexible reappraisals of social situations, facilitating more harmonious social interactions and prosocial behavior (Wolgast et al., 2020). Pishghadam et al. (2013) showed that avolved, exvolved and involved people have different interpretations of another person in an unfortunate predicament. Involved people’s ability of perspective-taking is higher than exvolved/avolved people’s, which can better promote individual’s prosocial behavior. The present study found that empathic concern could positively predict prosocial behaviors of college students in China, which is consistent with Balconi and Pozzoli’s (2009) research. When the object in a prosocial situation obviously needs help, individuals with a higher level of empathic concern are more likely to make decisions to help. That is, empathic concern is the capacity to “share in others’ feelings,” transforming others’ emotional representations into one’s own emotional representations (Belén and María, 2017); thus, it can predict individuals’ behavior response in prosocial situations.

We found that fantasy does not directly affect prosocial behavior, but it positively predicts individuals’ prosocial behavior through gratitude. Fantasy assesses the subject’s imaginative capacity by placing himself or herself in fictitious situations, trying to imagine how subject would feel in his/her situation (Mesurado and Richaud, 2017; Yalçın and DiPaola, 2019). According to Pishghadam et al. (2013), the frequency of sensory experience awakens and moves emotioncy to evoke emotions through the senses, which can relativize cognition. What’s more, an involved person has surpassed the exvolved and avolved people because the experience “provide an individual with a more thorough emotional experience of the object or concept” (Khoshsaligheh et al., 2018). So the level of individuals’ fantasy is different, when one’s fantasy level higher, one can better understand others’ misfortune or one’s own willingness to seek help. One can better perceive others’ goodwill and sacrifice for oneself or others with a higher level of gratitude, further promoting one’s prosocial behavior (Ma et al., 2017). Overall, fantasy empathy plays an essential role in forming and maintaining social interactions; it helps to coordinate actions, understand the intentions of others, and facilitate prosocial behavior between individuals (Omdahl, 1995; Bos and Stokes, 2019).

An important finding in our study is that the indirect effect of personal distress on prosocial behavior is negative, consistent with previous research (Batson et al., 1983; Carrera et al., 2013; Hortensius et al., 2016). This suggests that the effect of empathy is not always positive, and personal distress may decrease college students’ prosocial behavior. This may be explained by the fact that personal distress is a self-oriented emotion that evokes the egoistic motivation to reduce one’s own aversive arousal, directed to increasing one’s own welfare (egoistic). When the personal distress level is high, those oriented to alleviate their own discomfort will not help the unfortunate ones but instead tend to escape (i.e., physical or psychological escape) because they believe such discomfort will not last (Carrera et al., 2013).

Further, this study demonstrated that different components of empathy (perspective-taking, fantasy, empathic concern, and personal distress) were associated with prosocial behavior via gratitude. The present findings offer a new perspective and contribute to the broaden-and-build theory of positive emotions (Fredrickson, 2001, 2004) and psychological mediation framework of college students’ prosocial behaviors. According to this theory, gratitude may be an important mediator linking interpersonal factors to social adaptation. Gratitude is a positive emotional response to others’ kindness (Ma et al., 2017). In other words, when an individual’s empathy is activated, it is easy to perceive and experience the efforts and losses of others (De Waal, 2008), which will further stimulate the individual’s gratitude, thinking that the motivation behind the prosocial behavior is entirely altruistic (McCullough et al., 2002). It further stimulates the beneficiaries to engage in more helpful or altruistic behaviors (Tsang, 2006). Therefore, it is a more comprehensive and reasonable way to analyze the influence of empathy on prosocial behavior combined with gratitude.

### Practical Implications

Although affective empathy and cognitive empathy are independent of each other, they are closely related in function. The former is beneficial for individuals to generate prosocial motivation, while the latter is beneficial for individuals to choose effective ways to help others (Xiao et al., 2014). This reminds us that we should pay attention to the influence of different types of empathy on the prosocial behaviors of college students, improve their ability to empathize, and motivate them to engage in more prosocial behavior. However, for college students, personal distress is not always beneficial because it may decrease their prosocial behavior. Therefore, they should pay more attention to the gains and losses of others and decrease their personal distress through prosocial behavior instead of escaping when they find others in trouble. Moreover, in accordance with the broaden-and-build theory of positive emotions (Fredrickson, 2001), this study found that gratitude will motivate college students to engage in prosocial behavior. Therefore, it is necessary to emphasize the significance of gratitude education and cultivate college students’ gratitude awareness and mentality.

## Limitations and Future Research

There are some limitations despite the finding achieved. First of all, the current study was limited by its use of a cross-sectional design, preventing us from drawing any causal conclusions. Future research can combine ERP and fMRI techniques to further explore the differences in brain regions activated by different types of empathy in promoting prosocial behavior. Secondly, the study tested all variables through self-report measures, which may cause social desirability bias in the data, which can impact the accuracy of the results to a certain extent. Finally, the sample only contains China, which is not conducive to the promotion of the results. Future studies should use longitudinal design, combine self-reporting measures with experimental measures such as using projection tests and implicit experiments, consider the role of cultural differences, and conduct cross-cultural research to test the model of this study. Meanwhile, it is important to note that we also had limited statistical power to estimate some of the effects, and thus replication of these findings on personal distress with prosocial behaviors is needed.

## Data Availability Statement

The raw data supporting the conclusions of this article will be made available by the authors, without undue reservation.

## Ethics Statement

The studies involving human participants were reviewed and approved by Science and Technology Ethics Committee of the First Affiliated Hospital of Shihezi University School of Medicine (approval number: KJ2021-097-01). The patients/participants provided their written informed consent to participate in this study.

## Author Contributions

YP designed this study, performed the statistical analyses, and wrote the manuscript. CS revised the manuscript. CM provided constructive and editorial feedback on drafts of the manuscript. All authors contributed to the article and approved the submitted version.

## Conflict of Interest

The authors declare that the research was conducted in the absence of any commercial or financial relationships that could be construed as a potential conflict of interest.

## Publisher’s Note

All claims expressed in this article are solely those of the authors and do not necessarily represent those of their affiliated organizations, or those of the publisher, the editors and the reviewers. Any product that may be evaluated in this article, or claim that may be made by its manufacturer, is not guaranteed or endorsed by the publisher.
